# Supplementary material for: Health-Related Quality of Life during 26-Week Intervention with the New Nordic Renal Diet
Source: Nutrients. 2024 Jun 27;16(13):2038. doi: 10.3390/nu16132038 (PMC11243029; doi:10.3390/nu16132038)

**Supplementary material *(self-constructed 5-point Likert scale)***

*Health-related quality of life during 26-week intervention with the New Nordic Renal Diet*

## Likert scale – Health-related quality of life during the NNRD

| | |
|---|---|
| Study ID | ___________________________________ |
| How do you feel now,<br>compared to 6 months ago? | ○ Much better<br>○ In some scenarios slightly better<br>○ No change<br>○ Slightly worse<br>○ Much worse |
| How is your energy level now,<br>compared to 6 months ago? | ○ My energy level is much better now<br>○ My energy level is slightly better now<br>○ No change<br>○ It is slightly worse now<br>○ It is much worse now |
| How is your mood now,<br>compared to 6 months ago? | ○ My mood is much better now<br>○ My mood is slightly better now<br>○ No change<br>○ My mood is slightly worse now<br>○ My mood is much worse now |
| How is your stomach now,<br>compared to 6 months ago?<br>(E.g., pain, diarrhea, obstipation,<br>bloating etc.) | ○ I feel much better<br>○ On most days I feel slightly better now<br>○ No change<br>○ It is slightly worse now<br>○ It is much worse now |
| How is your quality of sleep now,<br>compared to 6 months ago? | ○ My sleep is much better now<br>○ Most days it is slightly better now<br>○ No change<br>○ It is slightly worse now<br>○ It is much worse now |
| How is the quality of your skin now,<br>compared to 6 months ago? | ○ It is much better now<br>○ In general, it is slightly better now<br>○ No change<br>○ It is slightly worse now<br>○ It is much worse now |

## Supplementary figure 1: Overview of Pearson’s correlations

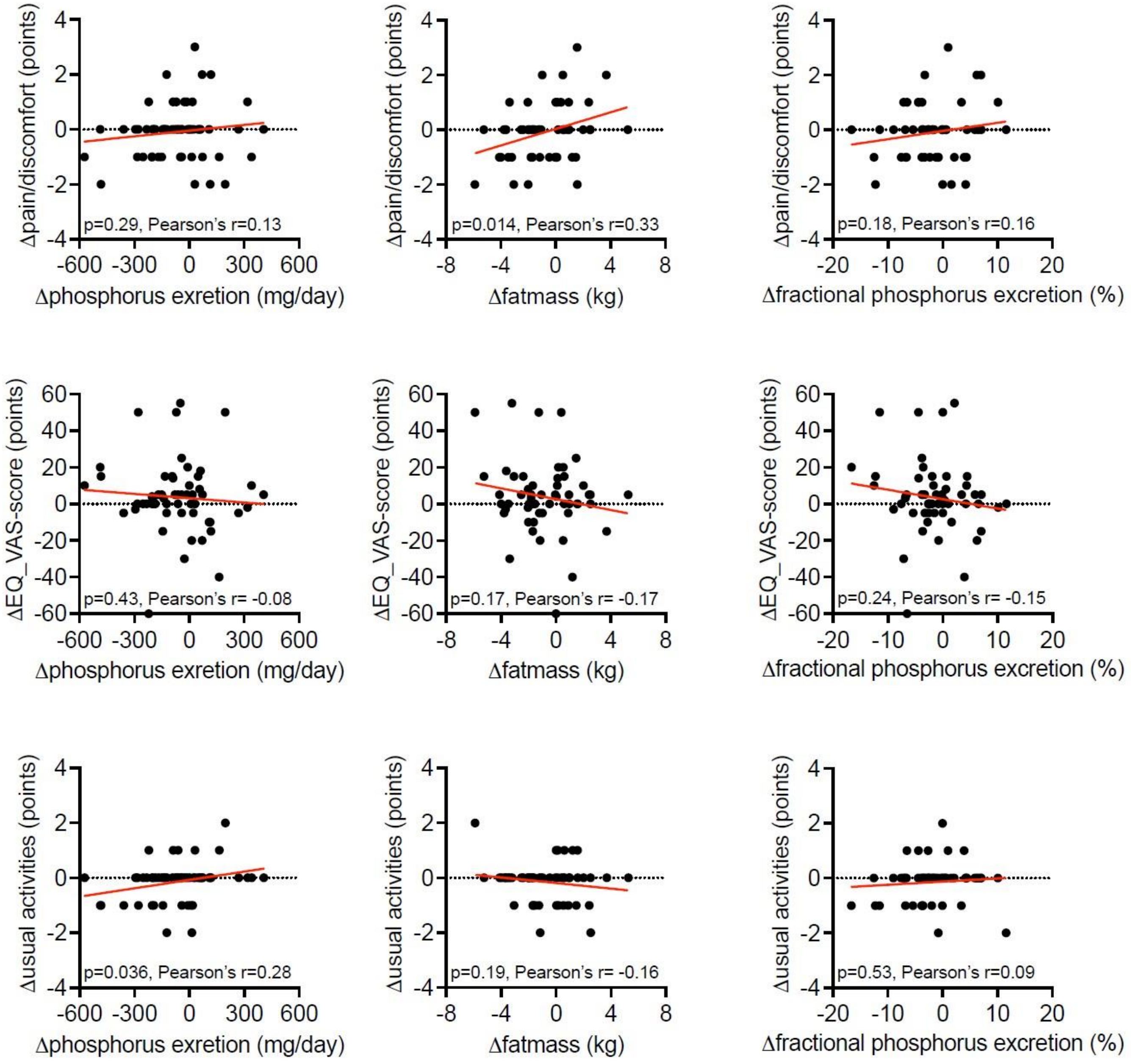

Supplement: Supplementary file 1 [file nutrients-16-02038-s001.zip › nutrients-3061439-supplementary.pdf]
